# Fault-Tolerant Control of a Variable-Pitch Quadrotor under Actuator Loss of Effectiveness and Wind Perturbations

**DOI:** 10.3390/s23104907

**Published:** 2023-05-19

**Authors:** Alessandro Baldini, Riccardo Felicetti, Alessandro Freddi, Andrea Monteriù

**Affiliations:** Department of Information Engineering, Università Politecnica delle Marche, 60131 Ancona, Italy; a.baldini@univpm.it (A.B.); r.felicetti@univpm.it (R.F.); a.monteriu@univpm.it (A.M.)

**Keywords:** variable-pitch quadrotors, fault-tolerant control, fault diagnosis, disturbance observer-based control, loss of effectiveness

## Abstract

The actuator fault-tolerant control problem for a variable-pitch quadrotor is addressed under uncertain conditions. Following a model-based approach, the plant nonlinear dynamics are faced with a disturbance observer-based control and a sequential quadratic programming control allocation, where only kinematic data of the onboard inertial measurement unit are required for the fault-tolerant control, i.e., it does not require the measurement of the motor speed nor the current drawn by the actuators. In the case of almost horizontal wind, a single observer handles both faults and the external disturbance. The estimation of the wind is fed forward by the controller, while the actuator fault estimation is exploited in the control allocation layer, which copes with the variable-pitch nonlinear dynamics, thrust saturation, and rate limits. Numerical simulations in the presence of measurement noise show the capability of the scheme to handle multiple actuator faults in a windy environment.

## 1. Introduction

Multirotor helicopters are a class of Unmanned Aerial Vehicle (UAV) that are employed in a wide range of applications, resulting in one of the most appealing classes of vehicles for both research and industry. The standard quadrotor represents the most common configuration, which is equipped with four fixed-pitch blades connected to independent motors. Variable-Pitch Quadrotors (VPQs) can instead vary both the rotation speed of each motor and the blade pitch of each propeller, theoretically doubling the degrees of freedom of each actuator. VPQs are also known as *collective pitch quadcopters* or *heliquads*.

This results in many advantages, from which it is possible to list higher thrust rate of change, reverse thrust, reverse flight capabilities, and scaling well with size [1,2,3]. Moreover, the popular ArduPilot control suite supports VPQs using the *Heliquad* configuration. Despite this, VPQs still represent a market niche, where few commercial devices (e.g., the Assault Reaper 500, the Stingray 500, and the WLtoys Assassin V383, for recreational use) are available. In most of the scientific literature, however, custom prototypes (e.g., [4,5,6]) are developed to test algorithms and control strategies. Fixed-pitch quadrotors and VPQs have similar dynamics, while relevant differences arise in the control effectiveness matrix. This allows for identical control laws for both configurations, but VPQs require a more complex control allocation algorithm. The additional degrees of freedom, provided by the pitch angles, can be exploited to satisfy additional constraints, such as minimizing energy consumption, handling the presence of faults and failures (as defined in [7]), etc.

Specifically, the control allocation algorithm, which manages the redundancy while respecting constraints, strictly depends on two key factors: the thrust model and the choice of the control inputs.

As regards the thrust model, several mathematical models have been proposed in the literature to catch the relation between motor speed, pitch angle, as well as propeller size and shape [3,5,6,8,9].

As for the choice of the control inputs, a VPQ instead has eight inputs available in general (i.e., four motor speeds and four pitch angles), a relevant input redundancy which is perfectly suited to provide actuator fault tolerance while satisfying actuation constraints. Two issues must be faced in this case. The increased number of actuators entails higher manufacturing costs and smaller payloads due to the extra weight. Moreover, the non-linearity of the control allocation problem makes it harder to solve online. In any case, the solution in which both the pitch angle and the motor speed can independently concur in providing the requested thrusts and torques is investigated in the literature, as carried out in [9,10] and, partially, in [6,11].

Alternatively, some simplifications have been proposed in the literature. The simpler solution is to assume constant motor speeds and use pitch angles as control inputs, as proposed in [1,3,8,12,13]; in this case, four inputs are available, so the allocation problem is very simple and there is no redundancy at all. Employing a single central motor to drive the four propellers makes a decrease in costs, weight, and inertia possible. However, assuming the rotational speed is constant is just a choice to simplify the control scheme, and employing a time-varying speed, even with a single central motor, is feasible (see, for example, [12,14]). Taking into account a variable centralized motor speed allows for some input redundancy (five inputs) and thus enables for fault-tolerant control, which is the focus of this paper.

Any fault that can affect a conventional quadrotor can affect VPQs as well, and they can involve any part of the rotor system (e.g., ESC, motor, propeller). The most common class of actuator fault is a Loss of Effectiveness (LOE), i.e., the produced thrust is lower than the expected one. In fact, LOE may be the consequence of many issues: battery voltage drop [15], increased drag due to the blade pitch [5], and of course any physical damage to the blades [16]. However, as for the specific case of fault diagnosis and tolerant control for VPQs, few works can be found in the literature. In [17], the presence of a pitch lock-in-place is considered for a VPQ, and both fault diagnosis and fault-tolerant control are developed. The authors of [14] focus on the effects of a lock-in-place in a centrally powered VPQ, but no fault diagnosis or fault-tolerant control are proposed. Finally, in [18], a fault detection and fault-tolerant control strategy to face both pitch lock-in-place and motor LOE is proposed.

A major issue in control of multirotors is the presence of wind. In the UAV literature, both active and passive fault-tolerant control schemes have been employed to cope with the presence of wind. In [19], the authors model the aerodynamic effects, define the upper bounds, and then deal with the wind by means of a robust sliding mode control; in [20], extended state observers are instead employed to mitigate the effects of wind. The presence of external disturbances is also detrimental to the fault diagnosis task, as the effects of both the disturbances and faults may trigger a fault detection algorithm. In this paper, we show that horizontal wind can be managed for both the purposes of fault diagnosis and disturbance rejection by means of a single observer that estimates the actuator faults and the effects of the wind.

The main contribution of this paper is proposing a novel, complete fault-tolerant control scheme for a VPQ under actuator faults and wind, which consists of three main tasks: fault and disturbance estimation, non-linear control with disturbance rejection, and fault-tolerant control allocation. The proposed control strategy fills a gap in the literature because, to the best of the authors’ knowledge, there are no fault-tolerant control schemes for VPQs that take into account common external disturbances such as the wind. With respect to the authors’ previous works [17,18], in this paper, we consider the effects of wind that introduce significant complexity in the first two tasks, i.e., fault estimation and control. As an additional contribution, we propose a generalized strategy that is well-suited for both centrally powered VPQs and VPQs with four independent motors. In fact, the fault and disturbance estimation strategy, as well as the non-linear control law, does not depend on the choice of the control inputs (i.e., centralized or independent propeller speed). On the other hand, the control allocation algorithm must exploit redundancy to achieve fault tolerance, so the choice of the control inputs is relevant; however, the proposed control allocation algorithm is designed such that it suffices to enable an equality constraint to deal with a centrally powered VPQ (instead of a conventional one with four separate motors).

The paper is structured as follows. In Section 2, the mathematical model of the vehicle is detailed, including the characterization of variable-pitch propellers and the model of actuator faults to be faced. Section 3 is devoted to the design of a Non-linear Disturbance Observer (NDO) to estimate faults and disturbances. In Section 4, a double-loop controller to track position and orientation, despite the presence of external disturbances, is introduced. In Section 5, a control allocation strategy that redistributes the control effort by taking into account the presence of actuator faults is detailed. Simulation results to show the effectiveness of the overall fault-tolerant control strategy are proposed in Section 6. Finally, conclusions and future works end the paper.

## 2. Mathematical Model

**Figure 1:**
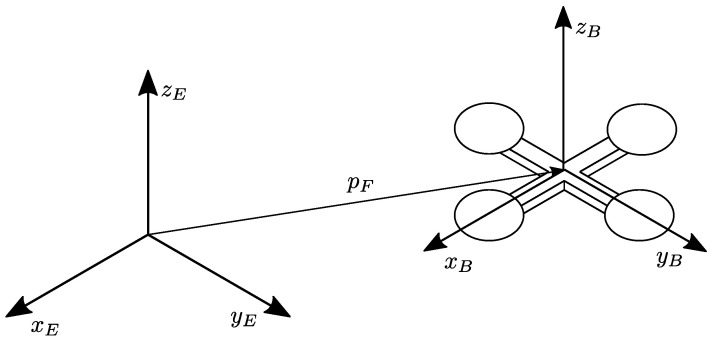
Quadrotor configuration and frames.

The overall kinematics and dynamics of a generic multirotor, thus including variable-pitch quadrotors, can be approximated as that of a unique rigid body with six degrees of freedom [21]. Let us consider an earth-fixed frame $R_{E}=\left(O_{E},x_{E},y_{E},z_{E}\right)$, which is assumed to be inertial, and a body-fixed frame $R_{B}=\left(O_{B},x_{B},y_{B},z_{B}\right)$, which is placed in the center of mass of the vehicle. For simplicity, we assume the center of mass coincides with the geometric center of the quadrotor, as depicted in Figure 1.

From now on, the standard basis vectors of $R^{3}$ are denoted with $e_{1},e_{2},e_{3}$. Let us denote with $p_{F}=\mathrm{col}\left(x_{F},y_{F},z_{F}\right)$ the position of the center of mass with respect to $R_{E}$, and with ω=col(p,q,r) the angular velocity of the vehicle. Neglecting the second-order effects (i.e., rotor dynamics, blade flapping, gyroscopic and inertial effects due to the rotors), the quadrotor kinematics and dynamics can be expressed as [21]

(1) $$\begin{array}{cc} m{\ddot{p}}_{F} & =-k_{t}{\dot{p}}_{F}-mge_{3}+RF_{m}^{B}+F_{w}^{E}\\ J\dot{\omega } & =-k_{r}\omega -\omega \times J\omega +\tau_{m}^{B}\\ \dot{\eta } & =T(\eta )\omega , \end{array}$$

where *m* is the total system mass, $J=\mathrm{diag}(J_{x},J_{y},J_{z})$ is the tensor of inertia along the $x_{B}$, $y_{B}$, and $z_{B}$ axes, $k_{t}$ and $k_{r}$ are the linear and angular friction coefficients, η=col(φ,θ,ψ) is the vector of the attitude angles (roll, pitch, and yaw, respectively) that characterize the rotation from $R_{B}$ to $R_{E}$.

(2) $$R=\left[\begin{array}{ccc} c_{\psi }\;c_{\theta }\; & \;c_{\psi }\;s_{\varphi }\;s_{\theta }-c_{\varphi }\;s_{\psi }\; & \;s_{\varphi }\;s_{\psi }+c_{\varphi }\;c_{\psi }\;s_{\theta }\\ c_{\theta }\;s_{\psi }\; & \;c_{\varphi }\;c_{\psi }+s_{\varphi }\;s_{\psi }\;s_{\theta }\; & \;c_{\varphi }\;s_{\psi }\;s_{\theta }-c_{\psi }\;s_{\varphi }\\ -s_{\theta }\; & \;c_{\theta }\;s_{\varphi }\; & \;c_{\varphi }\;c_{\theta } \end{array}\right],$$

where $\cos\left(\cdot \right)=c_{\left(\cdot \right)}$ and $\sin\left(\cdot \right)=s_{\left(\cdot \right)}$ are introduced for the sake of brevity, $F_{m}^{B}$ and $\tau_{m}^{B}$ are the overall force and torque due to the motors (decomposed in $R_{B}$), $F_{w}^{E}$ is the force due to the wind (decomposed in $R_{E}$), and T(η) is the kinematic coordinate transformation related to the adopted roll–pitch–yaw rotation, that is [22]:

(3) $$T\left(\eta \right)=\left[\begin{array}{ccc} 1 & \sin(\varphi )\tan(\theta ) & \cos(\varphi )\tan(\theta )\\ 0 & \cos(\varphi ) & -\sin(\varphi )\\ 0 & \frac{\sin(\varphi )}{\cos(\theta )} & \frac{\cos(\varphi )}{\cos(\theta )} \end{array}\right].$$

**Figure 2:**
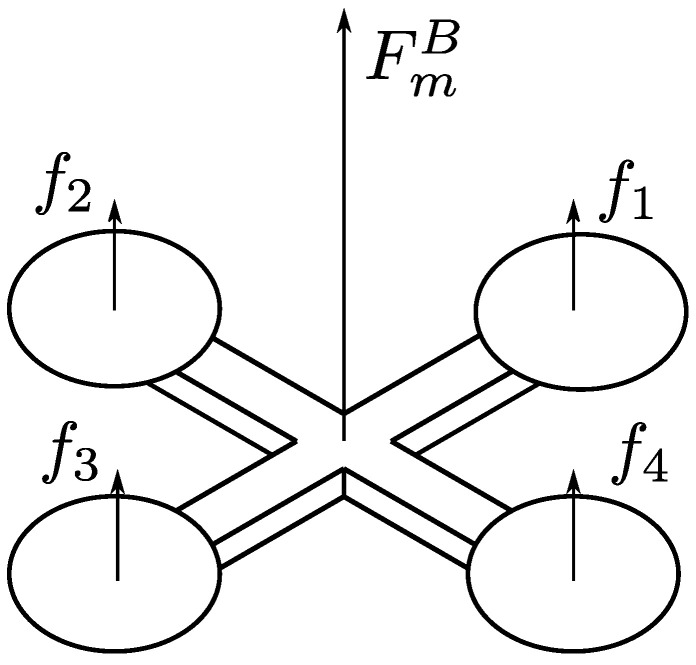
Propeller lift thrusts of the variable-pitch quadrotor.

Let us denote with $f_{i}\in R$ the actual lift thrust produced by the *i*-th actuator. In VPQs, as in conventional quadrotors, all the actuators are oriented upwards and they produce collinear lift forces only, as depicted in Figure 2.

Denoting with $u_{z}$ the overall lift force, given by $u_{z}=f_{1}+f_{2}+f_{3}+f_{4}$, we have

(4) $$\begin{array}{c} \begin{array}{cc} F_{m}^{B} & =\mathrm{col}(0,0,u_{z})\\ \tau_{m}^{B} & =\mathrm{col}(\tau_{p},\tau_{q},\tau_{r}). \end{array} \end{array}$$

In other words, the overall lift thrust is along $z_{B}$ only, because none of the actuators can generate a horizontal force in $R_{B}$. Then, substituting (4) in the first row of (1), we obtain the following model:

(5) $$\begin{array}{cc} m{\ddot{p}}_{F} & =-k_{t}{\dot{p}}_{F}-mge_{3}+u_{z}Re_{3}+F_{w}^{E}\\ J\dot{\omega } & =-k_{r}\omega -\omega \times J\omega +\tau_{m}^{B}\\ \dot{\eta } & =T(\eta )\omega . \end{array}$$

The four-dimensional vector $\mathrm{col}\left(u_{z},\tau_{m}^{B}\right)=\mathrm{col}\left(u_{z},\tau_{p},\tau_{q},\tau_{r}\right)$ is the so called virtual input vector.

### 2.1. Input Mapping

Consider the *i*-th motor. We denote with $f_{i}\in R$ the lift thrust generated by the propeller attached to the motor, with $d_{i}\in R$ the generated drag, and with $\Omega_{i}\in R$ the angular rate of the propeller, taken to be positive when the rotation of the motor matches that of the propeller (clockwise or counter-clockwise). Let us consider the case of rectangular, untwisted blades (which are suitable for variable-pitch drones) with uniform inflow. Let us assume the blade pitch is limited in order to avoid propeller stalling, which is an undesired effect to be avoided in practice. Then, both the lift force and drag of each rotor can be expressed as a function of the rotational speed [3]:

(6) $$\begin{array}{cc} f_{i} & =c_{L_{i}}\Omega_{i}^{2} \end{array}$$

(7) $$\begin{array}{cc} d_{i} & =c_{D_{i}}\Omega_{i}^{2}, \end{array}$$

where $c_{L_{i}}$ and $c_{D_{i}}$ are the lift and drag coefficients that depend on the blade geometry. In the case of fixed-pitch propellers, $c_{L_{i}}$ and $c_{D_{i}}$ are constant. In a variable-pitch propeller, however, as the pitch angle $\alpha_{i}\in R$ changes, the lift and drag coefficients change as well. Two main approaches to characterize the dependency of $c_{L_{i}}$ and $c_{D_{i}}$ on $\alpha_{i}$ can be found in [9], and they can be categorized into grey-box (e.g., [3]) and black-box (e.g., [5]) approaches. In this work, we follow the grey-box approach of [3], which makes use of the blade element theory and the momentum theory to obtain

(8) $$\begin{array}{cc} c_{L_{i}} & =c_{T_{i}}\left(\alpha_{i}\right)\rho \pi R_{a}^{4} \end{array}$$

(9) $$\begin{array}{cc} c_{D_{i}} & =c_{Q_{i}}\left(\alpha_{i}\right)\rho \pi R_{a}^{5}, \end{array}$$

where $R_{a}$ is the propeller radius, ρ is the air density, while $c_{T_{i}}\left(\cdot \right)$ and $c_{Q_{i}}\left(\cdot \right)$ are dimensionless lift and drag coefficients, which are functions of $\alpha_{i}$. Following [9,23], which assume steady-state linearized blade aerodynamics, we consider the implicit relations

(10) $$\begin{array}{cc} \alpha_{i} & =k_{1}c_{T_{i}}+k_{2}\sqrt{|c_{T_{i}}|} \end{array}$$

(11) $$\begin{array}{cc} c_{Q_{i}} & =k_{3}\sqrt{|c_{T_{i}}{|}^{3}}+k_{4}, \end{array}$$

where $k_{1}=\frac{6}{\sigma C_{l_{\alpha_{i}}}}$, $k_{2}=\frac{3}{2\sqrt{2}}$, $k_{3}=\frac{1}{\sqrt{2}}$, and $k_{4}=\frac{1}{8}\sigma C_{d0_{i}}$. In detail, $\sigma =\frac{N_{b}c}{\pi R_{a}}$ is the blade solidity, $N_{b}=2$ is the number of blades, *c* is the chord length, $C_{l_{\alpha_{i}}}$ is the 2D lift–curve–slope of the airfoil section, comprising the rotor, and finally, $C_{d0_{i}}$ is the zero-lift drag coefficient (see [9] for further details). Without loss of generality, we consider a ’+’ quadrotor configuration (see Figure 1), so the overall input mapping is

(12) $$\left[\begin{array}{c} u_{z}\\ \tau_{p}\\ \tau_{q}\\ \tau_{r} \end{array}\right]=\left[\begin{array}{c} c_{L_{1}}\left(\alpha_{1}\right)\Omega_{1}^{2}+c_{L_{2}}\left(\alpha_{2}\right)\Omega_{2}^{2}+c_{L_{3}}\left(\alpha_{3}\right)\Omega_{3}^{2}+c_{L_{4}}\left(\alpha_{4}\right)\Omega_{4}^{2}\\ -lc_{L_{2}}\left(\alpha_{2}\right)\Omega_{2}^{2}+lc_{L_{4}}\left(\alpha_{4}\right)\Omega_{4}^{2}\\ -lc_{L_{1}}\left(\alpha_{1}\right)\Omega_{1}^{2}+lc_{L_{3}}\left(\alpha_{3}\right)\Omega_{3}^{2}\\ -c_{D_{1}}\left(\alpha_{1}\right)\Omega_{1}^{2}+c_{D_{2}}\left(\alpha_{2}\right)\Omega_{2}^{2}-c_{D_{3}}\left(\alpha_{3}\right)\Omega_{3}^{2}+c_{D_{4}}\left(\alpha_{4}\right)\Omega_{4}^{2} \end{array}\right],$$

where *l* is the arm length. The mapping (12) is nonlinear in $\alpha_{i}$ and $\Omega_{i}$, which are the actual control inputs for each motor *i*, for a total of eight inputs.

### 2.2. Explicit Lift and Drag Functions

Equations (10) and (11) are implicit functions of $\alpha_{i}$, so they are not practical for online computation. In the literature, such functions are solved iteratively by means of numerical approaches [9]. In this section, instead, we introduce two equivalent equations that determine the coefficients $c_{T_{i}}$ and $c_{Q_{i}}$ in terms of explicit functions of $\alpha_{i}$.

The lift function $c_{T_{i}}\left(\alpha_{i}\right)$ in (10) is expected to be an odd function of $\alpha_{i}$ [9]. In fact, from (8), we obtain

(13) $$c_{T_{i}}\left(\alpha_{i}\right)=\frac{1}{k_{1}}\alpha_{i}+\frac{1}{2}\mathrm{sign}\left(\alpha_{i}\right)\left(\frac{k_{2}^{2}}{k_{1}^{2}}-\frac{k_{2}}{k_{1}^{2}}\sqrt{k_{2}^{2}+4k_{1}\left|\alpha_{i}\right|}\right),$$

which is an explicit odd function of $\alpha_{i}$. The drag function $c_{Q_{i}}\left(\alpha_{i}\right)$ in (11) is expected to be an even function of $\alpha_{i}$ [9]. Then, noting that $c_{T_{i}}\left(\right|\alpha_{i}\left|\right)=sign\left(\alpha_{i}\right)c_{T}\left(\alpha_{i}\right)$, (11) can be rewritten as

(14) $$c_{Q_{i}}\left(\alpha_{i}\right)=k_{3}\sqrt{sign\left(\alpha_{i}\right)c_{T_{i}}{\left(\alpha_{i}\right)}^{3}}+k_{4},$$

which is an explicit even function of $\alpha_{i}$. We also calculate in advance the derivatives of (13) and (14) with respect to $\alpha_{i}$, as they are going to be employed in the remainder of the paper. We have

(15) $$\begin{array}{cc} c_{T_{i}}^{\prime }\left(\alpha_{i}\right) & =\frac{1}{k_{1}}-\frac{k_{2}}{k_{1}}\frac{1}{\sqrt{k_{2}^{2}+4k_{1}\left|\alpha_{i}\right|}}\\ c_{Q_{i}}^{\prime }\left(\alpha_{i}\right) & =\frac{3}{2}k_{3}c_{T_{i}}^{\prime }\left(\alpha_{i}\right)sign\left(\alpha_{i}\right)\sqrt{sign\left(\alpha_{i}\right)c_{T_{i}}\left(\alpha_{i}\right)}\\ c_{L_{i}}^{\prime }\left(\alpha_{i}\right) & =c_{T_{i}}^{\prime }\left(\alpha_{i}\right)\rho \pi R_{a}^{4}\\ c_{D_{i}}^{\prime }\left(\alpha_{i}\right) & =c_{Q_{i}}^{\prime }\left(\alpha_{i}\right)\rho \pi R_{a}^{5}, \end{array}$$

where the second row is obtained with the use of

(16) $$\frac{dc_{T_{i}}\left(\right|\alpha_{i}\left|\right)}{d\alpha_{i}}=sign\left(\alpha_{i}\right)\frac{dc_{T_{i}}\left(\alpha_{i}\right)}{d\alpha_{i}}.$$

### 2.3. Actuator Faults and Control Input Definition

A total of eight control inputs are considered, which are the pitch angles $\alpha_{i}$ and the squares of the desired rotor speeds, denoted as $u_{i}$, for i=1,…,4. All the control inputs are collapsed in the vectors

(17) $$\begin{array}{c} \begin{array}{cc} \alpha & =\mathrm{col}(\alpha_{1},\alpha_{2},\alpha_{3},\alpha_{4}),\\ u & =\mathrm{col}(u_{1},u_{2},u_{3},u_{4}). \end{array} \end{array}$$

In the absence of faults, the relation $\Omega_{i}^{2}=u_{i}$ holds. For each actuator *i*, a possible LOE is considered, so that the resulting angular speed may be lower than the desired one, i.e.,

(18) $$\begin{array}{c} \Omega_{i}^{2}=w_{i}u_{i}. \end{array}$$

Each $w_{i}$, for i=1,…,4, is an unknown and time-varying quantity which models the fault magnitude on the *i*-th actuator. It is constrained in $w_{i}\in \left[0,1\right]$, where $w_{i}=1$ corresponds to the faultless scenario. For the sake of brevity, the fault magnitudes are collected in the vector

(19) $$\begin{array}{c} w=\mathrm{col}(w_{1},w_{2},w_{3},w_{4}). \end{array}$$

## 3. Wind and Fault Estimation

We assume the wind acts on the $x_{E}-y_{E}$ plane only, that is, $F_{w}^{E}=f_{wx}e_{1}+f_{wy}e_{2}$. In other words, we neglect the updraft, and we claim that the horizontal wind does not generate any significant torque due to the symmetry of the vehicle. Thus, six target independent variables need to be estimated; these are $f_{wx},f_{wy}$ and $w_{1},w_{2},w_{3},w_{4}$, and they are summarized in the extended uncertainty vector $\bar{w}=\mathrm{col}\left(f_{wx},f_{wy},w_{1},w_{2},w_{3},w_{4}\right)$. By handling the joint vector $\bar{w}$ as an external disturbance, a disturbance observer for the estimation $\hat{\bar{w}}$ of $\bar{w}$ is then designed, which in our purpose can be defined as a dynamical system in the form

(20) $$\begin{array}{cc} \dot{\hat{s}} & =F(\hat{s},\bar{x},\bar{u}) \end{array}$$

(21) $$\begin{array}{cc} \hat{\bar{w}} & =H(\hat{s},\bar{x},\bar{u}), \end{array}$$

for which $∥\hat{\bar{w}}-\bar{w}∥\to 0$ as t→∞, and where $\hat{s}$ is the observer state, $\bar{x}=\mathrm{col}\left(p_{F},{\dot{p}}_{F},\omega ,\eta \right)$ and $\bar{u}=\mathrm{col}\left(u,\alpha \right)$ compactly refers to the quadrotor state and the control inputs, and where F(·) and H(·) are smooth function to design. Several disturbance observers have been proposed in the literature. Here, we follow the NDO proposed in [24], which can be summarized as follows.

1. Define an auxiliary variable *s* which directly depends on the estimation target $\bar{w}$. Usually, the auxiliary variable $s=\bar{w}-\lambda \left(\bar{x},\bar{u}\right)$ is chosen, for some smooth function λ(·).
2. Find the dynamics $\dot{s}$ of the auxiliary variable *s*. In this step, the disturbance observer design problem is turned into a state observer design problem.
3. Based on the auxiliary dynamics $\dot{s}$, design a state observer for the estimation $\hat{s}$ of *s*.
4. Define $\hat{\bar{w}}$ by using the inverse function of the chosen auxiliary variable definition.

For design purposes, the model is conveniently rewritten in Section 3.1. Then, the design of the NDO is proposed in Section 3.2.

**Remark 1.**  *The estimation of the actuator faults and the external force generated by the wind is obtained by the disturbance observer, where only kinematic data are required, i.e., without the need of measuring motor speeds, motor currents, etc.*

**Remark 2.**  *The design of λ(·) involves the solution of a set of partial differential equations.*

**Remark 3.**  *If the dynamics of $\bar{w}$ is uncertain, the ultimate boundedness of $∥\hat{\bar{w}}-\bar{w}∥$ is demanded instead of convergence.*

### 3.1. Model Manipulation

Note that the input mapping (12) and the action of the fault (18) can be rewritten as

(22) $$\left[\begin{array}{c} u_{z}\\ \tau_{p}\\ \tau_{q}\\ \tau_{r} \end{array}\right]=\left[\begin{array}{cccc} c_{L_{1}}\left(\alpha_{1}\right) & c_{L_{2}}\left(\alpha_{2}\right) & c_{L_{3}}\left(\alpha_{3}\right) & c_{L_{4}}\left(\alpha_{4}\right)\\ 0 & -lc_{L_{2}}\left(\alpha_{2}\right) & 0 & lc_{L_{4}}\left(\alpha_{4}\right)\\ -lc_{L_{1}}\left(\alpha_{1}\right) & 0 & lc_{L_{3}}\left(\alpha_{3}\right) & 0\\ -c_{D_{1}}\left(\alpha_{1}\right) & c_{D_{2}}\left(\alpha_{2}\right) & -c_{D_{3}}\left(\alpha_{3}\right) & c_{D_{4}}\left(\alpha_{4}\right) \end{array}\right]\left[\begin{array}{c} \Omega_{1}^{2}\\ \Omega_{2}^{2}\\ \Omega_{3}^{2}\\ \Omega_{4}^{2} \end{array}\right]≜B_{c}\left(\alpha \right)\left[\begin{array}{c} \Omega_{1}^{2}\\ \Omega_{2}^{2}\\ \Omega_{3}^{2}\\ \Omega_{4}^{2} \end{array}\right]$$

and

(23) $$\left[\begin{array}{c} \Omega_{1}^{2}\\ \Omega_{2}^{2}\\ \Omega_{3}^{2}\\ \Omega_{4}^{2} \end{array}\right]=\left[\begin{array}{cccc} w_{1} & 0 & 0 & 0\\ 0 & w_{2} & 0 & 0\\ 0 & 0 & w_{3} & 0\\ 0 & 0 & 0 & w_{4} \end{array}\right]\left[\begin{array}{c} u_{1}\\ u_{2}\\ u_{3}\\ u_{4} \end{array}\right]=\mathrm{diag}\left(w\right)u=\mathrm{diag}\left(u\right)w,$$

resulting in the overall relation $\mathrm{col}\left(u_{z},\tau_{m}^{B}\right)=B_{c}\left(\alpha \right)\mathrm{diag}\left(u\right)w$.

The quadrotor kinematics and dynamics in (5) can be equivalently expressed as

(24) $$\frac{d}{dt}\left[\begin{array}{c} p_{F}\\ \eta \end{array}\right]=\left[\begin{array}{c} {\dot{p}}_{F}\\ T(\eta )\omega \end{array}\right]$$

(25) $$\frac{d}{dt}\left[\begin{array}{c} {\dot{p}}_{F}\\ \omega \end{array}\right]=\left[\begin{array}{c} -\frac{k_{t}}{m}{\dot{p}}_{F}-ge_{3}\\ -k_{r}J^{-1}\omega -J^{-1}\left(\omega \times J\omega \right) \end{array}\right]+\frac{1}{m}\left[\begin{array}{cc} e_{1} & e_{2}\\ 0 & 0 \end{array}\right]\left[\begin{array}{c} f_{wx}\\ f_{wy} \end{array}\right]+\left[\begin{array}{cc} \frac{1}{m}Re_{3} & 0\\ 0 & J^{-1} \end{array}\right]\left[\begin{array}{c} u_{z}\\ \tau_{m}^{B} \end{array}\right].$$

Then, defining $z_{1}=\mathrm{col}\left(p_{F},\eta \right)$ and $z_{2}=\mathrm{col}\left({\dot{p}}_{F},\omega \right)$, we can rewrite the overall quadrotor model in order to show that the non-linear model is affine with respect to the uncertainty $\bar{w}$. In fact, we have

(26) $$\begin{array}{cc} {\dot{z}}_{1} & =h_{1}\left(z_{1},z_{2}\right)\\ {\dot{z}}_{2} & =h_{2}\left(z_{2}\right)+P\left(z_{1},u,\alpha \right)\bar{w}, \end{array}$$

where

(27) $$h_{1}\left(z_{1},z_{2}\right)=\left[\begin{array}{c} {\dot{p}}_{F}\\ T(\eta )\omega \end{array}\right],\;\;h_{2}\left(z_{2}\right)=\left[\begin{array}{c} -\frac{k_{t}}{m}{\dot{p}}_{F}-ge_{3}\\ -k_{r}J^{-1}\omega -J^{-1}\left(\omega \times J\omega \right) \end{array}\right],$$

and $P\left(z_{1},u,\alpha \right)=\left[\begin{array}{cc} P_{1} & P_{2}\left(z_{1},u,\alpha \right) \end{array}\right]\in R^{6\times 6}$, with

(28) $$P_{1}=\frac{1}{m}\left[\begin{array}{cc} e_{1} & e_{2}\\ 0 & 0 \end{array}\right]\in R^{6\times 2},\;P_{2}\left(z_{1},u,\alpha \right)=\left[\begin{array}{cc} \frac{1}{m}Re_{3} & 0\\ 0 & J^{-1} \end{array}\right]B_{c}\left(\alpha \right)\mathrm{diag}\left(u\right)\in R^{6\times 4}.$$

Finally, *P* is almost everywhere nonsingular, since

(29) $$\begin{array}{cc} \det(P) & =\frac{1}{m^{2}}\det\left(\left[\begin{array}{cc} \frac{1}{m}\cos\left(\varphi \right)\cos\left(\theta \right) & 0\\ 0 & J^{-1} \end{array}\right]B_{c}\left(\alpha \right)\mathrm{diag}\left(u\right)\right)\\ \; & =u_{1}u_{2}u_{3}u_{4}\frac{\cos(\varphi )\cos(\theta )}{m^{3}J_{x}J_{y}J_{z}}\det\left(B_{c}\left(\alpha \right)\right), \end{array}$$

where $\det(B_{c}\left(\alpha \right))\neq 0$ almost everywhere (assuming l>0). Noting that each $u_{i}$ is positive in real flight conditions, cos(φ)cos(θ) is different from zero in non-aerobatic flight, and $\det(B_{c}\left(\alpha \right))\neq 0$, then the inverse of *P* is well-defined in practical flight conditions.

### 3.2. Disturbance Observer Design

The NDO is designed on model (26). Indeed, the NDO design in DOBC is well-known for control affine systems [25], so the idea is actually based on the affine uncertainty, which is $\bar{w}$ in our problem. Consider the auxiliary variable $s=\bar{w}-\lambda \left(z_{1},z_{2},u,\alpha \right)$, where λ(·) is a function to design. For the sake of brevity, we denote $\lambda =\lambda (z_{1},z_{2},u,\alpha )$. By differentiation, we have

(30) $$\begin{array}{cc} \dot{s} & =\dot{\bar{w}}-\frac{\partial \lambda }{\partial z_{1}}h_{1}\left(z_{1},z_{2}\right)-\frac{\partial \lambda }{\partial z_{2}}\left(h_{2}\left(z_{2}\right)+P\left(z_{1},u,\alpha \right)\bar{w}\right)-\frac{\partial \lambda }{\partial u}\dot{u}-\frac{\partial \lambda }{\partial u}\dot{\alpha }\\ \; & =\dot{\bar{w}}-\frac{\partial \lambda }{\partial z_{1}}h_{1}\left(z_{1},z_{2}\right)-\frac{\partial \lambda }{\partial z_{2}}\left(h_{2}\left(z_{2}\right)+P\left(z_{1},u,\alpha \right)\left(s+\lambda \right)\right)-\frac{\partial \lambda }{\partial u}\dot{u}-\frac{\partial \lambda }{\partial u}\dot{\alpha }\\ \; & =-\frac{\partial \lambda }{\partial z_{2}}P\left(z_{1},u,\alpha \right)s+R_{s}+\dot{\bar{w}}, \end{array}$$

where $R_{s}$ is a known term given by

(31) $$R_{s}=-\frac{\partial \lambda }{\partial z_{1}}h_{1}\left(z_{1},z_{2}\right)-\frac{\partial \lambda }{\partial z_{2}}\left(h_{2}\left(z_{2}\right)+P\left(z_{1},u,\alpha \right)\lambda \right)-\frac{\partial \lambda }{\partial u}\dot{u}-\frac{\partial \lambda }{\partial u}\dot{\alpha }.$$

The disturbance observer is then designed as

(32) $$\begin{array}{cc} \dot{\hat{s}} & =-\frac{\partial \lambda }{\partial z_{2}}P\left(z_{1},u,\alpha \right)\hat{s}+R_{s} \end{array}$$

(33) $$\begin{array}{cc} \hat{\bar{w}} & =\hat{s}+\lambda , \end{array}$$

where $\hat{s}$ and $\hat{\bar{w}}$ are estimations for *s* and $\bar{w}$, respectively. The function λ(·) is investigated through the estimation error dynamics. On this purpose, let us consider the error variables $\tilde{s}=s-\hat{s}$ and $\tilde{\bar{w}}=\bar{w}-\tilde{\bar{w}}$. The error dynamics is

(34) $$\begin{array}{cc} \dot{\tilde{s}} & =-\frac{\partial \lambda }{\partial z_{2}}P\left(z_{1},u,\alpha \right)\hat{s}+\dot{\bar{w}} \end{array}$$

(35) $$\begin{array}{cc} \tilde{\bar{w}} & =\tilde{s}. \end{array}$$

With the choice $\lambda \left(z_{1},z_{2},u,\alpha \right)=HP{\left(z_{1},u,\alpha \right)}^{-1}z_{2}$, where −H is Hurwitz, the overall estimation error boils down to

(36) $$\dot{\tilde{\bar{w}}}=-H\tilde{\bar{w}}+\dot{\bar{w}},$$

which is an asymptotically stable linear time-invariant system, perturbed by the unknown input $\dot{\bar{w}}$.

## 4. Control Law Design

A classical inner/outer loop approach is employed to calculate the desired virtual inputs [26,27], where the small angle approximation is adopted (φ≈0 and θ≈0). Moreover, in order to avoid a conflict between the inner and the outer loop, a spectral separation is required; to achieve this, in practice, a higher rate is employed for the inner loop with respect to the outer loop [28].

### 4.1. Outer Loop Design

Given the time-varying position references $x_{F_{r}}$ and $y_{F_{r}}$ (for $x_{F}$ and $y_{F}$, respectively), the outer loop is designed to calculate the references $\varphi_{r}$ and $\theta_{r}$ (for the angles φ and θ, respectively) in order to track the position reference. Exploiting the small angle approximation [26,27], that is, φ≈0 and θ≈0, we have

(37) $$\left[\begin{array}{c} e_{1}^{T}\\ e_{2}^{T} \end{array}\right]Re_{3}\approx \left[\begin{array}{cc} \sin(\psi ) & \cos(\psi )\\ -\cos(\psi ) & \sin(\psi ) \end{array}\right]\left[\begin{array}{c} \varphi \\ \theta \end{array}\right]≜R_{2}\left(\psi \right)\left[\begin{array}{c} \varphi \\ \theta \end{array}\right].$$

Substituting (37) in (5), it follows that

(38) $$\begin{array}{cc} \left[\begin{array}{c} {\ddot{x}}_{F}\\ {\ddot{y}}_{F} \end{array}\right] & =\left[\begin{array}{c} e_{1}^{T}\\ e_{2}^{T} \end{array}\right]{\ddot{p}}_{F}=-\frac{k_{t}}{m}\left[\begin{array}{c} {\dot{x}}_{F}\\ {\dot{y}}_{F} \end{array}\right]+\frac{u_{z}}{m}\left[\begin{array}{c} e_{1}^{T}\\ e_{2}^{T} \end{array}\right]Re_{3}+\frac{1}{m}\left[\begin{array}{c} f_{wx}\\ f_{wy} \end{array}\right]\\ \; & \approx -\frac{k_{t}}{m}\left[\begin{array}{c} {\dot{x}}_{F}\\ {\dot{y}}_{F} \end{array}\right]+\frac{u_{z}}{m}R_{2}\left(\psi \right)\left[\begin{array}{c} \varphi \\ \theta \end{array}\right]+\frac{1}{m}\left[\begin{array}{c} f_{wx}\\ f_{wy} \end{array}\right]. \end{array}$$

As the inner loop runs at a higher rate with respect to the outer loop, we can assume the inner loop is fast enough to track $\varphi_{r}$ and $\theta_{r}$ [26]. Hence, by solving (38) for the variables φ and θ, we obtain the ideal references $\varphi_{r}$ and $\theta_{r}$ for $u_{z}\neq 0$, that is,

(39) $$\left[\begin{array}{c} \varphi_{r}\\ \theta_{r} \end{array}\right]=\frac{m}{u_{z}}R_{2}^{T}\left(\varphi \right)\left(\frac{k_{t}}{m}\left[\begin{array}{c} {\dot{x}}_{F}\\ {\dot{y}}_{F} \end{array}\right]-\frac{1}{m}\left[\begin{array}{c} {\hat{f}}_{wx}\\ {\hat{f}}_{wy} \end{array}\right]+\left[\begin{array}{c} v_{x}\\ v_{y} \end{array}\right]\right),$$

where $v_{x}$ and $v_{y}$ are injection inputs, defined as

(40) $$\begin{array}{cc} v_{x} & ={\ddot{x}}_{Fr}-\alpha_{x1}\left({\dot{x}}_{F}-{\dot{x}}_{Fr}\right)-\alpha_{x0}\left(x_{F}-x_{Fr}\right) \end{array}$$

(41) $$\begin{array}{cc} v_{y} & ={\ddot{y}}_{Fr}-\alpha_{y1}\left({\dot{y}}_{F}-{\dot{y}}_{Fr}\right)-\alpha_{y0}\left(y_{F}-y_{Fr}\right). \end{array}$$

The parameters $\alpha_{x1},\alpha_{x0},\alpha_{y1},\alpha_{y0}$ are chosen according to the desired pole placement problem. In fact, if the angles φ and θ follow references (39)–(41), we can show by substitution in (5) that the outer loop tracking error dynamics becomes:

(42) $$\begin{array}{cc} {\ddot{e}}_{x}+\alpha_{x1}{\dot{e}}_{x}+\alpha_{x_{0}}e_{x} & =\frac{1}{m}{\tilde{f}}_{wx} \end{array}$$

(43) $$\begin{array}{cc} {\ddot{e}}_{y}+\alpha_{y1}{\dot{e}}_{x}+\alpha_{y_{0}}e_{y} & =\frac{1}{m}{\tilde{f}}_{wy}, \end{array}$$

where $e_{x}=x_{F}-x_{Fr}$, $e_{y}=y_{F}-y_{Fr}$, ${\tilde{f}}_{wx}=f_{wx}-{\hat{f}}_{wx}$ and ${\tilde{f}}_{wy}=f_{wy}-{\hat{f}}_{wy}$.

### 4.2. Inner Loop Design

Let $z_{Fr}$ and $\psi_{r}$ be the reference signals for $z_{F}$ and ψ that we want to track, and let $\varphi_{r}$ and $\theta_{r}$ be the reference signals for φ and θ (provided by the outer loop in (39)). From (5), it follows that

(44) $$\begin{array}{cc} {\ddot{z}}_{F} & =-\frac{k_{t}}{m}{\dot{z}}_{F}-g+\frac{u_{z}}{m}\cos\left(\varphi \right)\cos\left(\theta \right) \end{array}$$

(45) $$\begin{array}{cc} \ddot{\eta } & =\dot{T}\left(\eta \right)\omega -k_{r}T\left(\eta \right)J^{-1}\omega -T\left(\eta \right)J^{-1}\left(\omega \times J\omega \right)+T\left(\eta \right)J^{-1}\tau_{m}^{B}, \end{array}$$

so we can set the reference values $u_{z,r}$ and $\tau_{m,r}^{B}$ (for $u_{z}$ and $\tau_{m}^{B}$, respectively) as

(46) $$\begin{array}{c} \begin{array}{cc} u_{z,r} & =\frac{m}{\cos(\varphi )\cos(\theta )}\left(\frac{k_{t}}{m}{\dot{z}}_{F}+g+v_{z}\right)\\ \tau_{m,r}^{B} & =JT{\left(\eta \right)}^{-1}\left(-\dot{T}\left(\eta \right)\omega +k_{r}T\left(\eta \right)J^{-1}\omega +T\left(\eta \right)J^{-1}\left(\omega \times J\omega \right)+v_{\eta }\right). \end{array} \end{array}$$

The terms $v_{z}\in R$ and $v_{\eta }\in R^{3}$ are auxiliary inputs, and they are set as

(47) $$\begin{array}{cc} v_{z} & ={\ddot{z}}_{Fr}-\alpha_{z1}\left({\dot{z}}_{F}-{\dot{z}}_{Fr}\right)-\alpha_{z0}\left(z_{F}-z_{Fr}\right) \end{array}$$

(48) $$\begin{array}{cc} v_{\eta } & ={\ddot{\eta }}_{r}-\alpha_{\eta 1}\left(T\left(\eta \right)\omega -{\dot{\eta }}_{r}\right)-\alpha_{\eta 0}\left(\eta -\eta_{r}\right), \end{array}$$

where the scalars $\alpha_{z1},\alpha_{z0}$ and the matrices $\alpha_{\eta 1},\alpha_{\eta 0}$ are set according to the desired pole placement. If the variables $u_{z}$ and $\tau_{m}^{B}$ follow references (46)–(48), we can show by substitution in (5) that the inner loop tracking error dynamics becomes

(49) $$\begin{array}{cc} {\ddot{e}}_{z}+\alpha_{z1}{\dot{e}}_{z}+\alpha_{z_{0}}e_{z} & =0 \end{array}$$

(50) $$\begin{array}{cc} {\ddot{e}}_{\eta }+\alpha_{\eta 1}{\dot{e}}_{\eta }+\alpha_{\eta_{0}}e_{\eta } & =0, \end{array}$$

where $e_{z}=z_{F}-z_{Fr}$ and $e_{\eta }=\eta -\eta_{r}$.

## 5. Control Allocation

In this section, we show how the virtual input (i.e., $u_{z}$ and $\tau_{m}^{B}$) can be distributed among the motors (i.e., *u*) and the pitch servos (i.e., α). Let us consider a non-linear optimization problem in the optimization variables α and *u*:

(51) $$\begin{array}{cc} \min_{\alpha ,u} & \;J(\alpha ,u) \end{array}$$

(52) $$\begin{array}{cc} s.t. & \;h(\alpha ,u)=0 \end{array}$$

(53) $$\begin{array}{cc} \; & \;g(\alpha ,u)\leq 0, \end{array}$$

where J(α,u) in (51) is a cost function to be determined, while (52) and (53) represent generic non-linear and nonconvex constraints. In the literature, the control allocation problem in multirotors is usually tackled with lightweight methods, such as pseudo-inverse and redistributed pseudo-inverse [29,30]. Such methods are motivated by the need for high rates, but they are not effective in the presence of constraints and rate limits. Moreover, they need a significant simplification of the problem formulation (i.e., linear or quadratic cost functions, linear equality constraints, potentially also removing inequality constraints). In the following, we set up the control allocation problem and we detail a method to solve it efficiently by means of a locally convex Quadratic Programming (QP) reformulation (as carried out, for example, in [31] for a marine vessel).

The method that is detailed in this section to solve the non-linear allocation problem (51)–(53) represents a simplified version of the generic Sequential Quadratic Programming (SQP) strategy (see, for example, [32]) to solve non-linear optimization problems. The main differences are

- SQP employs the Hessian of the Lagrangian in the cost function, while we employ the Hessian of the original cost function. In other words, we always set the Lagrange multipliers’ initial guess to zero for simplicity, so that the Hessian of the Lagrangian coincides with the Hessian of the original cost function.
- Once the search direction is found, a full step is performed in the proposed method, and no merit function is computed.
- The stopping criterion in the proposed method boils down to performing just a single iteration (i.e., a single QP is solved as in Real-Time Iteration [33], instead of a sequence of QP problems as done in SQP), and so no convergence criteria are needed.

These modifications introduce some loss of accuracy in the solution, but they reduce the number of evaluations, in order to perform the allocation faster in view of online implementation.

### 5.1. Equality Constraints

The equality constraints (52) originate from (22)–(23), namely

(54) $$h(\alpha ,u)=B\left[\begin{array}{c} \mathrm{diag}\left(w\right)\mathrm{diag}({\bar{c}}_{L}\left(\alpha \right))u\\ \mathrm{diag}\left(w\right)\mathrm{diag}({\bar{c}}_{D}\left(\alpha \right))u \end{array}\right]-\left[\begin{array}{c} u_{z}\\ \tau_{m}^{B} \end{array}\right]$$

(55) $$\;\;\;\;=B\left[\begin{array}{c} \mathrm{diag}\left(w\right)\mathrm{diag}\left(u\right){\bar{c}}_{L}\left(\alpha \right)\\ \mathrm{diag}\left(w\right)\mathrm{diag}\left(u\right){\bar{c}}_{D}\left(\alpha \right) \end{array}\right]-\left[\begin{array}{c} u_{z}\\ \tau_{m}^{B} \end{array}\right]=0,$$

where

(56) $$B=\left[\begin{array}{cccccccc} 1 & 1 & 1 & 1 & 0 & 0 & 0 & 0\\ 0 & -l & 0 & l & 0 & 0 & 0 & 0\\ -l & 0 & l & 0 & 0 & 0 & 0 & 0\\ 0 & 0 & 0 & 0 & -1 & 1 & -1 & 1 \end{array}\right]$$

and

(57) $$\begin{array}{c} \begin{array}{cc} {\bar{c}}_{L}\left(\alpha \right) & =\mathrm{col}(c_{L}\left(\alpha_{1}\right),c_{L}\left(\alpha_{2}\right),c_{L}\left(\alpha_{3}\right),c_{L}\left(\alpha_{4}\right))\\ {\bar{c}}_{D}\left(\alpha \right) & =\mathrm{col}(c_{D}\left(\alpha_{1}\right),c_{D}\left(\alpha_{2}\right),c_{D}\left(\alpha_{3}\right),c_{D}\left(\alpha_{4}\right))\\ {\bar{c}}_{L}^{\prime }\left(\alpha \right) & =\mathrm{col}(c_{L}^{\prime }\left(\alpha_{1}\right),c_{L}^{\prime }\left(\alpha_{2}\right),c_{L}^{\prime }\left(\alpha_{3}\right),c_{L}^{\prime }\left(\alpha_{4}\right))\\ {\bar{c}}_{D}^{\prime }\left(\alpha \right) & =\mathrm{col}(c_{D}^{\prime }\left(\alpha_{1}\right),c_{D}^{\prime }\left(\alpha_{2}\right),c_{D}^{\prime }\left(\alpha_{3}\right),c_{D}^{\prime }\left(\alpha_{4}\right)). \end{array} \end{array}$$

Please note that $c_{L}\left(\alpha_{i}\right)$ and $c_{D}\left(\alpha_{i}\right)$ have been defined in (8), while $c_{L}^{\prime }\left(\alpha_{i}\right)$ and $c_{D}^{\prime }\left(\alpha_{i}\right)$ have been defined in (15). The time-varying numeric values for $u_{z}$, $\tau_{m}^{B}$, and *w* in (54) are given by (46) ($u_{z,r}$, $\tau_{m,r}^{B}$) and (33) ($\hat{\bar{w}}$), respectively; in other words, the actual lift force and torques must satisfy those required by the inner loop, also taking into account the actual fault estimation.

Please note that the equality constraints are non-linear and nonconvex with respect to the optimization variables α and *u*. Non-linear optimization problems are hard to solve online due to computational limits, as well as because the existence of the solution is not guaranteed in general. To solve efficiently the problem online, the equality constraints (52) are linearized, as in SQP, so that a QP problem can be obtained. Using the first-order Taylor approximation around $\left(\alpha_{0},u_{0}\right)$, we have

(58) $$h\left(\alpha ,u\right)\approx h\left(\alpha_{0},u_{0}\right)+\frac{\partial h(\alpha_{0},u_{0})}{\partial \alpha }\left(\alpha -\alpha_{0}\right)+\frac{\partial h(\alpha_{0},u_{0})}{\partial u}\left(u-u_{0}\right),$$

where $\alpha_{0}$ and $u_{0}$ represent the current value of α and *u*, respectively. Then, (54) can be approximated by

(59) $$\underset{A_{eq}}{\underset{︸}{\left[\frac{\partial h(\alpha_{0},u_{0})}{\partial \alpha }\;\frac{\partial h(\alpha_{0},u_{0})}{\partial u}\right]}}\underset{x}{\underset{︸}{\left[\begin{array}{c} \Delta \alpha \\ \Delta u \end{array}\right]}}=\underset{b_{eq}}{\underset{︸}{-h(\alpha_{0},u_{0})}},$$

where $\Delta \alpha =\alpha -\alpha_{0}$, $\Delta u=u-u_{0}$, and

(60) $$\begin{array}{c} \begin{array}{cc} \frac{\partial h(\alpha_{0},u_{0})}{\partial \alpha } & =B\left[\begin{array}{c} \mathrm{diag}\left(w\right)\mathrm{diag}\left(u_{0}\right)\mathrm{diag}\left(c_{L}^{\prime }\right)\\ \mathrm{diag}\left(w\right)\mathrm{diag}\left(u_{0}\right)\mathrm{diag}\left(c_{D}^{\prime }\right) \end{array}\right]\\ \frac{\partial h(\alpha_{0},u_{0})}{\partial u} & =B\left[\begin{array}{c} \mathrm{diag}\left(w\right)\mathrm{diag}({\bar{c}}_{L}\left(\alpha_{0}\right))\\ \mathrm{diag}\left(w\right)\mathrm{diag}({\bar{c}}_{D}\left(\alpha_{0}\right)) \end{array}\right]\\ h(\alpha_{0},u_{0}) & =B\left[\begin{array}{c} \mathrm{diag}\left(w\right)\mathrm{diag}\left({\bar{c}}_{L}\left(\alpha_{0}\right)\right)u_{0}\\ \mathrm{diag}\left(w\right)\mathrm{diag}\left({\bar{c}}_{D}\left(\alpha_{0}\right)\right)u_{0} \end{array}\right]-\left[\begin{array}{c} u_{z}\\ \tau_{m}^{B} \end{array}\right]. \end{array} \end{array}$$

As *w* is not available, to build the control allocation problem online we replace it in (60) with its estimation contained in $\hat{\bar{w}}$.

**Remark 4.**  *In the case of a centralized motor, it suffices to add the constraint $\Delta u_{1}=\Delta u_{2}=\Delta u_{3}=\Delta u_{4}$; provided that the same initial value is employed for u, then $u_{1}=u_{2}=u_{3}=u_{4}$ holds. In other words, (59) is replaced by*

(61) $$\underset{A_{eq}}{\underset{︸}{\left[\begin{array}{cc} \frac{\partial h(\alpha_{0},u_{0})}{\partial \alpha } & \frac{\partial h(\alpha_{0},u_{0})}{\partial u}\\ \begin{array}{cccc} 0 & 0 & 0 & 0\\ 0 & 0 & 0 & 0\\ 0 & 0 & 0 & 0 \end{array} & \begin{array}{cccc} 1 & -1 & 0 & 0\\ 0 & 1 & -1 & 0\\ 0 & 0 & 1 & -1 \end{array} \end{array}\right]}}\underset{x}{\underset{︸}{\left[\begin{array}{c} \begin{array}{c} \Delta \alpha \\ \Delta u \end{array} \end{array}\right]}}=\underset{b_{eq}}{\underset{︸}{\left[\begin{array}{c} -h(\alpha_{0},u_{0})\\ 0\\ 0\\ 0 \end{array}\right]}}$$

### 5.2. Inequality Constraints

The inequality constraints of interest to be included in (53), in practice, are saturation constraints and rate limits, i.e.,

(62) $$\begin{array}{c} \begin{array}{cc} \underline{\alpha } & \leq \alpha \leq \bar{\alpha }\\ \underline{u} & \leq u\leq \bar{u}\\ \underline{\Delta \alpha } & \leq \alpha -\alpha_{0}\leq \bar{\Delta \alpha }\\ \underline{\Delta u} & \leq u-u_{0}\leq \bar{\Delta u}. \end{array} \end{array}$$

Such inequality constraints are affine with respect to the optimization variables α and *u*, so they are tractable in online optimization.

Replacing (52) with its approximation (59) (or (61), for a single centralized motor) and expressing the constraints (62) in the variable x=col(Δα,Δu), we obtain an optimization problem in the form

(63) $$\begin{array}{c} \begin{array}{cc} \min_{x} & \;J(x)\\ s.t. & \;A_{eq}x=b_{eq}\\ \; & \;\underline{x}\leq x\leq \bar{x}, \end{array} \end{array}$$

where

(64) $$\begin{array}{c} \begin{array}{c} \underline{x}=\mathrm{col}\left(\max\left(\underline{\alpha }-\alpha_{0},\underline{\Delta \alpha }\right)\;,\;\max\left(\underline{u}-u_{0},\underline{\Delta u}\right)\right)\\ \bar{x}=\mathrm{col}\left(\min\left(\bar{\alpha }-\alpha_{0},\bar{\Delta \alpha }\right)\;,\;\min\left(\bar{u}-u_{0},\bar{\Delta u}\right)\right) \end{array} \end{array}$$

are defined to express the inequality constraints (62) in a more compact way. Please note that (63) is a QP problem by construction, provided that the cost function J(x) is quadratic.

### 5.3. Cost Function

The main term to be considered in a cost function for UAVs, especially in the case of multirotors, is the energy consumption E(x). A quadratic form for E(x) is often assumed in the UAV literature [29,30]; such a choice is mainly motivated by simplicity, as it usually leads to a QP problem. Motivated by the need for autonomy in UAVs, more detailed representations, coming from physically accurate consumption models, have been proposed in the literature as well. The most employed models are second-order polynomials [9] and irrational functions [34], as well as more generic non-linear functions [6]. All of the previous choices fit well with the proposed framework, as it is always possible to locally approximate the cost function with a quadratic cost function, as done for example in [31]. For the purpose of this work, the steady-state thruster consumption from [34] is employed. Given a generic matrix $M=\left[m_{ij}\right]\in R^{m\times n}$ and k∈R, let us introduce, with a slight abuse of notation, the short ${\left|M\right|}^{k}=\left[\right|m_{ij}{|}^{k}]$ (i.e., the *k*-th power of the absolute values of the entries of *M*, in an element-wise fashion). Then, the steady-state thruster consumption for a quadrotor can be rewritten as

(65) $$E\left(x\right)=\sum_{i=1}^{4}q_{u,i}{\left|u_{i}\right|}^{3/2}=|u^{T}{|}^{3/4}Q_{u}{\left|u\right|}^{3/4},$$

where ${\left|u\right|}^{3/4}$ and $|u^{T}{|}^{3/4}$ should be intended element-wise as already anticipated. Furthermore, $q_{u,i}$ is assumed to be constant for simplicity (see [34] for further details) and

(66) $$Q_{u}=\mathrm{diag}\left(q_{u,1},\ldots ,q_{u,4}\right),$$

with $q_{u,i}>0$ for i=1,…,4. Hence, we can define the cost function

(67) $$J\left(x\right)=E\left(x\right)+\Delta \alpha^{T}Q_{\Delta \alpha }\Delta \alpha +\Delta u^{T}Q_{\Delta u}\Delta u+{\left(\alpha -\alpha_{p}\right)}^{T}Q_{\alpha }\left(\alpha -\alpha_{p}\right),$$

where $Q_{\Delta \alpha }$, $Q_{\Delta u}$, $Q_{\alpha }$, $Q_{u}$ are symmetric positive definite matrices. The first term of (67), which takes into account energy consumption, is detailed in (65). The second and the third quadratic terms of (67) discourage variations in the control inputs to minimize actuator wear and tear. The last term of (67) defines preferred values $\alpha_{p}$ for the pitch angles α. This term becomes necessary in order to compensate for the first term (i.e., the one that discourages energy consumption), because the actuator is more energy-efficient when the pitch angle is large in magnitude. Thus, in the absence of the fourth term, the pitch angle is often stuck to its saturation limit to minimize energy consumption. In such a case, the only way to increase the lift further is to increase the motor speed, as the pitch angle cannot be increased in magnitude, so the maneuverability of the vehicle worsens.

Considering the second-order Taylor approximation for E(x), i.e.,

(68) $$E\left(x\right)\approx |u_{0}^{T}{|}^{3/4}Q_{u}|u_{0}{|}^{3/4}+\frac{3}{2}{\left|u_{0}^{T}\right|}^{1/2}Q_{u}\Delta u+\frac{3}{8}\Delta u^{T}Q_{u}\mathrm{diag}\left(|u_{0}{|}^{-1/2}\right)\Delta u,$$

and performing some manipulation, (67) becomes

(69) $$\begin{array}{c} \begin{array}{cc} J(x)= & \frac{1}{2}x^{T}\underset{H}{\underset{︸}{\left[\begin{array}{cc} 2(Q_{\alpha }+Q_{\Delta \alpha }) & 0_{4\times 4}\\ 0_{4\times 4} & 2Q_{\Delta u}+\frac{3}{4}Q_{u}\mathrm{diag}\left(|u_{0}{|}^{-1/2}\right) \end{array}\right]}}x\\ \; & +\underset{f^{T}}{\underset{︸}{\left[\begin{array}{cc} 2{\left(\alpha_{0}-\alpha_{p}\right)}^{T}Q_{\alpha } & \frac{3}{2}{\left(|u_{0}{|}^{1/2}\right)}^{T}Q_{u} \end{array}\right]}}x+c, \end{array} \end{array}$$

where *c* is a constant term (i.e., a term that does not depend on *x*), which is irrelevant for the solution of the optimization problem, so it can be neglected.

### 5.4. Scaling and Infeasibility

The problem formulation according to (59) (or (61), for a single centralized motor), (64), (69), that is,

(70) $$\begin{array}{c} \begin{array}{cc} \min_{x} & \;\frac{1}{2}x^{T}Hx+f^{T}x\\ s.t. & \;A_{eq}x=b_{eq}\\ \; & \;\underline{x}\leq x\leq \bar{x}, \end{array} \end{array}$$

shows two drawbacks. First of all, the existence of a feasible solution is not guaranteed, so problem infeasibility must be dealt with. Moreover, the problem is ill-conditioned, because the magnitude of Δα and Δu is very different; according to (17)–(18), α is typically in the range of decimals (in radians), while *u* is typically in the range of tens of thousands (in radians per second squared), and the same occurs for Δα and Δu.

To improve the problem conditioning, diagonal scaling is performed [32]. We define a new optimization variable

(71) $$\begin{array}{c} z=P^{-1}x, \end{array}$$

where $P=\mathrm{diag}(\max(|\underline{x}\left|,\right|\bar{x}\left|)\right)$ is a diagonal matrix such that the components of *z* share the same magnitude, as they become constrained in [−1,1] according to the rate limits in (62) (please note that the box constraint estimation [−1,1] is conservative; tighter constraints may apply).

In order to deal with infeasible problems, we soften the equality constraint by adding a vector of unconstrained artificial variables ζ into the equality constraint, while minimizing the norm of the artificial variable. The overall problem (70), after the change of variables and the softening of equality constraints, becomes

(72) $$\begin{array}{c} \begin{array}{cc} \min_{z,\zeta } & \;\frac{1}{2}z^{T}P^{T}HPz+f^{T}Pz+\zeta^{T}Q_{\zeta }\zeta \\ s.t. & \;A_{eq}Pz+\zeta =b_{eq}\\ \; & \;P^{-1}\underline{x}\leq z\leq P^{-1}\bar{x}, \end{array} \end{array}$$

which can be finally rewritten in the classical form that is usually required by QP solvers, i.e.,

(73) $$\begin{array}{c} \begin{array}{cc} y^{*}=\arg\min_{y} & \;\frac{1}{2}y^{T}\overset{ˇ}{H}y+{\overset{ˇ}{f}}^{T}y\\ s.t. & \;{\overset{ˇ}{A}}_{eq}y={\overset{ˇ}{b}}_{eq}\\ \; & \;\overset{ˇ}{A}y\leq \overset{ˇ}{b}\\ \; & \;\underline{y}\leq y\leq \bar{y}, \end{array} \end{array}$$

where

(74) $$\begin{array}{c} \begin{array}{cccc} \begin{array}{c} y=\left[\begin{array}{c} z\\ \zeta \end{array}\right] \end{array} & \overset{ˇ}{H}=\left[\begin{array}{cc} P^{T}HP & 0_{8x4}\\ 0_{4x8} & Q_{\zeta } \end{array}\right] & {\overset{ˇ}{f}}^{T}=\left[\begin{array}{cc} f^{T}P & 0_{1x4} \end{array}\right]\\ {\overset{ˇ}{A}}_{eq}=\left[\begin{array}{cc} A_{eq}P & I_{4} \end{array}\right] & {\overset{ˇ}{b}}_{eq}=b_{eq} & \underline{y}=\left[\begin{array}{c} P^{-1}\underline{x}\\ \underline{\zeta } \end{array}\right] & \bar{y}=\left[\begin{array}{c} P^{-1}\bar{x}\\ \bar{\zeta } \end{array}\right], \end{array} \end{array}$$

$\underline{\zeta }$ and $\bar{\zeta }$ are large enough to guarantee feasibility (e.g., $\bar{\zeta }=-\underline{\zeta }=M$, M→∞), while $\overset{ˇ}{A}$ and $\overset{ˇ}{b}$ are empty matrices, because no inequality constraints are left, with an exception made for the upper and lower bounds that are usually introduced separately.

Once the solution $y^{*}$ of (72)–(74) is calculated, the solution to the control allocation problem is

(75) $$\begin{array}{c} \begin{array}{cc} x^{*} & =\left[\begin{array}{c} \Delta \alpha^{*}\\ \Delta u^{*} \end{array}\right]=\left[\begin{array}{cc} P & 0_{8x4} \end{array}\right]y^{*}\\ \alpha^{*} & =\alpha_{0}+\Delta \alpha^{*}\\ u^{*} & =u_{0}+\Delta u^{*}. \end{array} \end{array}$$

**Remark 5.**  *The choice of a quadratic penalty for ζ is made for simplicity, as it introduces a small number of additional variables and it makes the cost function smooth and hence easier to solve online. This strategy is similar to the quadratic penalty method in [32] and, more precisely, to the weighted least squares in [35]. As a drawback, such a penalty term is not exact, i.e., the solution of the penalized problem is not equivalent to the solution of the original problem [32,36]. In particular, the higher the penalty through $Q_{\zeta }$, the more the solutions of the two problems coincide, but also the worse is the conditioning. Thus, $Q_{\zeta }$ is a trade-off between ideal solution quality and acceptable conditioning. We claim that this issue is not crucial, because several approximations are introduced to simplify the problem, and so the solution is not exact anyway. Alternatively, it is possible to introduce additional positively constrained slack variables and to employ exact penalty functions, such as $\ell_{1}$ and $\ell_{\infty }$ penalties, at the price of dealing with the nonsmoothness of the cost function (see [32] for further details).*

## 6. Numerical Simulation

A numerical simulation is performed using MATLAB, where the inner loop and the outer loop run at 400 Hz (i.e., ArduPilot’s loop rate) and 10 Hz, respectively. A zero-order hold is performed between consecutive samples. The quadrotor parameters are set according to [18] and they are reported in Table 1. Different from [18], the friction is neglected and the thrust-to-weight ratio is slightly increased (from 2.5 to 3.131) in order to face severe faults.

The control parameters were set empirically through a coarse grid search (as such, better performances could be achieved with a finer tuning). The closed-loop poles for the inner loop are placed in −2 and −2.1 for $z_{F}$, in −10 and −11 for φ and θ, and finally in −5 and −5.1 for ψ. The outer loop poles are instead placed in −1 and −2 for both $x_{F}$ and $y_{F}$. A properly scaled white Gaussian sensor noise is simulated according to an MPU-9250 IMU TDK InvenSense [37], which is a common device in quadrotor flight controllers (i.e., in Pixhawk 4).

### 6.1. Independent Motors’ Speed

**Figure 3:**
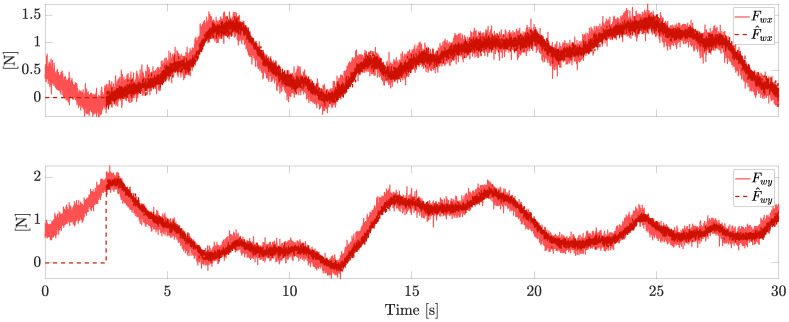
Horizontal wind estimation (independent motors’ speed).

Let us first consider a VPQ equipped with four independent motors and four independent pitch servos. The simulation is performed for a total of 30 s under the external wind force reported in Figure 3, which also reports the correspondent estimations provided by the observer, where the gain matrix has been set as H=5I, with *I* being the identity matrix.

Due to the stochastic nature of the wind, achieving the boundedness of the estimation error replaces the ideal goal of a perfect convergence, which is here achieved in the case of bounded derivatives of the wind force. The estimation of the faults and the wind is practically started after a finite time from the beginning of the flight (i.e., after 1s of simulation in this example), in order to make the initial conditions of the observer practically irrelevant.

**Figure 4:**
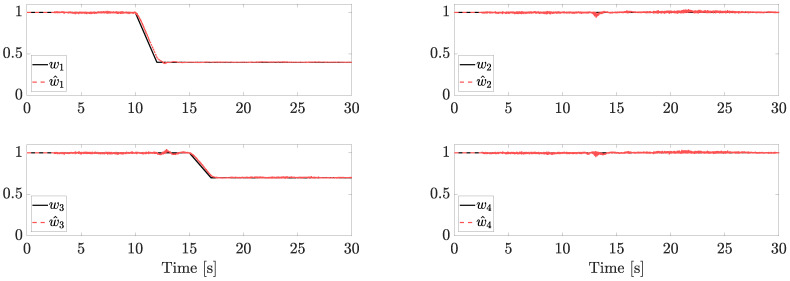
Fault estimation (independent motors’ speed).

Faults components and their estimations are reported in Figure 4.

Two trapezoidal faults are injected. The first one affects motor 1 after 10 s (the LOE magnitude is 60%, i.e., $w_{1}=0.4$) and a second one affects motor 3 after 15 s (the LOE magnitude is 30%, i.e., $w_{3}=0.7$). In the range t∈[0,1], each ${\hat{w}}_{i}$ is set equal to 1, matching the reasonable assumption of no faults at the initial time (or, without loss of generality, we assume that we know the fault status at the beginning of the flight). Note that, since each estimation is provided independently from the NDO, the same results can be achieved in the case of simultaneous faults.

**Figure 5:**
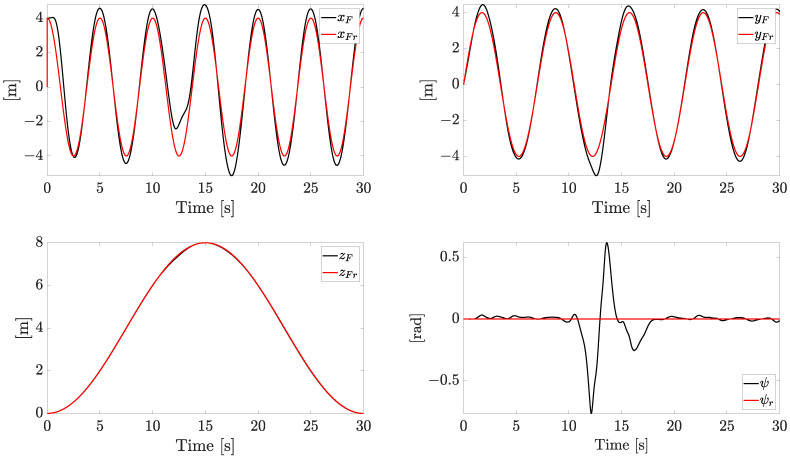
Tracking performances (independent motors’ speed).

The tracking reference components and the related state variables are reported in Figure 5.

The references on $x_{F}$ and $y_{F}$ are sinusoidal signals, the reference on $z_{F}$ forces the quadrotor to face both ascending and descending profiles, while the yaw angle is kept at zero. The system is forced to change the velocity constantly in both magnitude and direction, with a linear speed magnitude up to 7 m/s. All the references are smoothly tracked with an acceptable error (the plotted variables are the actual state variables instead of the noisy ones), with the exception of the yaw component (ψ), which exhibits a temporary increasing error when the fault worsens.

**Figure 6:**
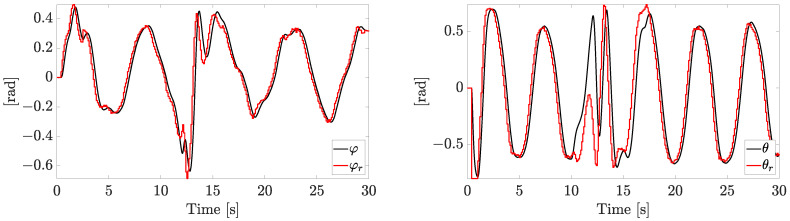
Inner loop tracking performances on desired angles (independent motors’ speed).

The references $\varphi_{r}$ and $\theta_{r}$ generated by the outer loop are reported in Figure 6.

Please note that, when the fault is rapidly worsening, the tracking capabilities in the inner loop are affected. The inner/outer loop approach, which employs a higher frequency of the inner loop and a zero-order hold, allows us to track the references, despite the presence of noise, faults, and disturbances.

**Figure 7:**
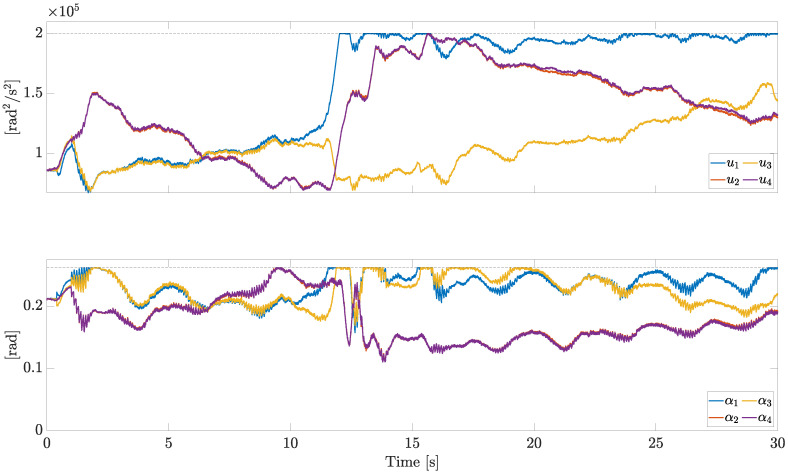
Control inputs *u* and α (independent motors’ speed).

The control inputs *u* and α are reported in Figure 7.

All the actuators are subject to the same saturation constraints. This allows us to plot the bounds with horizontal dashed lines in Figure 7 (upper bounds in this case). In particular, the saturation limits have been set as

(76) $$\begin{array}{c} \begin{array}{cc} \underline{\alpha } & =\mathrm{col}(\alpha_{min},\alpha_{min},\alpha_{min},\alpha_{min})\\ \bar{\alpha } & =\mathrm{col}(\alpha_{max},\alpha_{max},\alpha_{max},\alpha_{max})\\ \underline{u} & =\mathrm{col}(u_{min},u_{min},u_{min},u_{min})\\ \bar{u} & =\mathrm{col}(u_{max},u_{max},u_{max},u_{max})\\ \overset{-}{\Delta \alpha } & =-\underline{\Delta \alpha }=\mathrm{col}\left(\Delta \alpha_{max},\Delta \alpha_{max},\Delta \alpha_{max},\Delta \alpha_{max}\right)\\ \overset{-}{\Delta u} & =-\underline{\Delta u}=\mathrm{col}\left(\Delta u_{max},\Delta u_{max},\Delta u_{max},\Delta u_{max}\right), \end{array} \end{array}$$

where $u_{min}$, $u_{max}$, $\alpha_{min}$, and $\alpha_{max}$ are detailed in Table 1. Considering $\alpha =\bar{\alpha }$, $\bar{u}$ corresponds to a thrust-to-weight ratio equal to 3.131.

At the beginning, when no fault is present, the opposite actuators (1 and 3, 2 and 4) show similar motor speeds and pitch angles, due to symmetry. As soon as the fault on motor 1 occurs, the opposite actuators 1 and 3 behave differently; in response to the severe fault acting on motor 1, the control allocation algorithm demands a high speed and a large pitch angle to compensate for the missing lift force.

### 6.2. Centralized Motor

**Figure 8:**
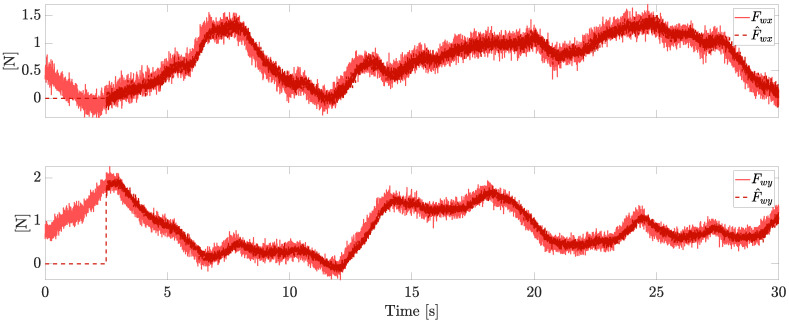
Horizontal wind estimation (centralized motor).

In this section, we consider a VPQ equipped with a single centralized motor and four independent pitch servos. The simulation conditions and the wind components are the same as the previous simulation, including the same random seed. Figure 8 reports the wind effect and its estimations provided by the observer; no noticeable differences can be noted, as expected, because the fault estimation does not directly depend on the actual inputs.

**Figure 9:**
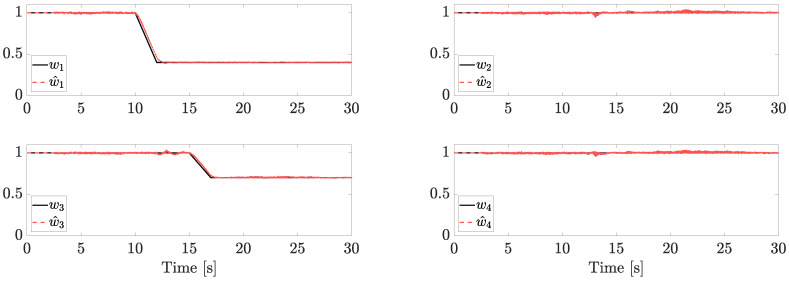
Fault estimation (centralized motor).

The same consideration holds for the faults and their estimations (Figure 9).

**Figure 10:**
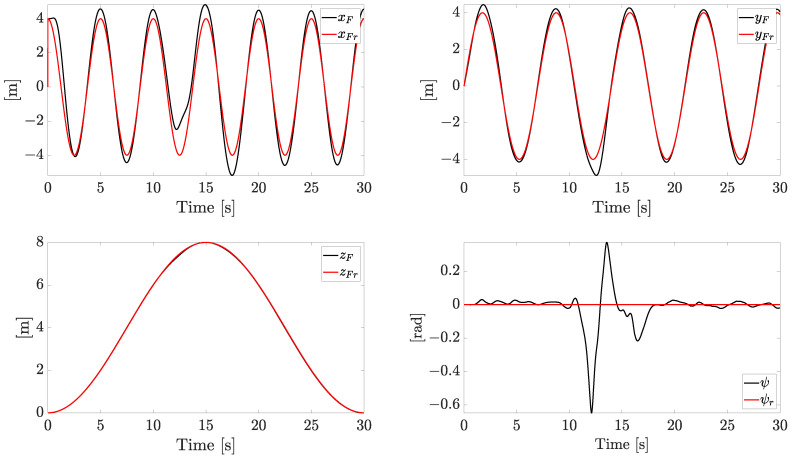
Tracking performances (centralized motor).

**Figure 11:**
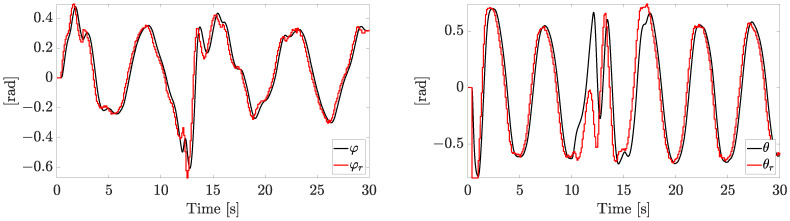
Inner loop tracking performances on desired angles (centralized motor).

The tracking reference components and the related state variables are reported in Figure 10 and Figure 11. The performances are similar; minor differences in the outer loop tracking capabilities can be detected.

**Figure 12:**
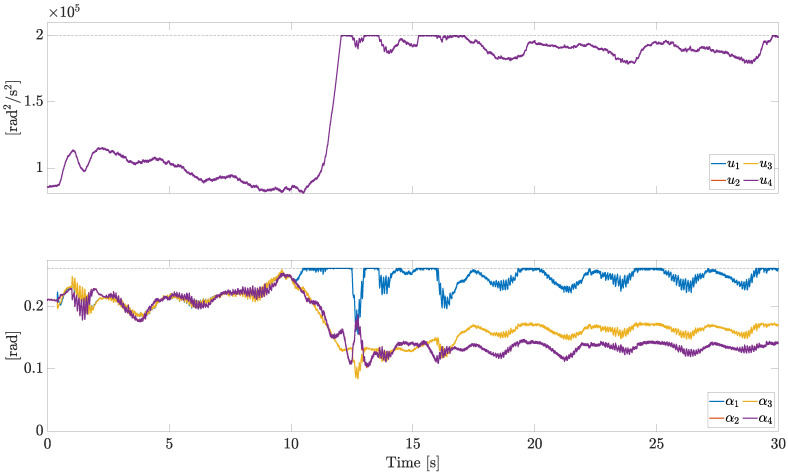
Control inputs *u* and α (centralized motor).

Finally, the control inputs *u* and α are reported in Figure 12.

First of all, please note that the motor speeds coincide, as expected. Furthermore, in response to the fault acting on motor 1, the control allocation algorithm demands a high motor speed to compensate for the missing lift force. As there is only one centralized motor, the speed of every propeller must increase. Analogously, please note that, the larger the fault, the higher the average value of the pitch angle.

As the motor speed is higher on average in the case of the centralized motor (Figure 7 and Figure 12), the energy consumption is higher. This is unavoidable, because in the case of the centralized motor, the input redundancy is lower than in the case of independent motors. In Table 2, we summarize the overall normalized (i.e., $Q_{u}=I$) consumption during the flight, as proposed in (65).

## 7. Conclusions

Unlike standard quadrotors that are equipped with four fixed-pitch blades and four independent motors, VPQs can vary both the rotation speed and the blade pitch of each propeller, thus increasing the degrees of freedom for control purposes. In addition to higher thrust rate of change, reverse thrust and reverse flight capabilities, and scaling well with size, VPQs enable for fault-tolerant control strategies thanks to their inherent input redundancy, whether they are equipped with four independent motors or a centralized one. We have shown that a single observer can estimate both actuator loss of effectiveness and the external disturbance given by horizontal wind. According to the disturbance observer-based control framework, the estimation of the wind is fed forward in the outer (position) control loop. The estimation of the actuator loss of effectiveness is instead employed in the control allocation algorithm. It manages each propeller’s pitch and motor speed to generate the force and the torques commanded by the inner (attitude and altitude) control loop. Simulation results show that the estimation of both the disturbance and the actuator loss of effectiveness is practically feasible in the presence of conventional measurement noise in commercial devices. We remark that the fault estimation is performed from kinematic data of the onboard inertial measurement unit only, without the need to measure the motor speed nor the current drawn by the actuators.

Comparing centralized and independent motors, the former is less efficient in terms of energy consumption, especially in the case of faults, because all the propellers must spin faster. Clearly, many other factors should be considered to evaluate the energy consumption properly, such as possible weight and cost reduction. In any case, both centralized and independent motors can tolerate actuator faults using the proposed scheme. The proposed control scheme could also deal with pitch lock-in-place, provided that reliable information on the stuck fault is available (e.g., measuring the current pitch angle or estimating it through a fault detection algorithm). Practically, it is sufficient to constrain the faulty pitch servo to the current value, and the control allocation algorithm can accommodate for the stuck fault using the remaining control inputs. However, in the case of a single centralized motor, the reduced amount of redundancy makes it less robust to lock-in-place faults and severe loss of effectiveness.

## Figures and Tables

**Table 1:** Variable-pitch quadrotor parameters.

Parameter	Variable	Value	Unit
Total system mass	*m*	1.37	[kg]
Inertia about $x_{B}$, $y_{B}$	$J_{x}$,$J_{y}$	7.5 × 10${}^{-3}$	[kg· m${}^{2}$]
Inertia about $z_{B}$	$J_{z}$	1.3 × 10${}^{-2}$	[kg· m${}^{2}$]
Arm length	*l*	0.3	[m]
Lift curve slope	$c_{l_{\alpha }}$	5.23	[−]
Zero lift drag coefficient	$c_{d_{0}}$	0.01	[−]
Propeller radius	$R_{a}$	0.18	[m]
Propeller chord	*c*	0.03	[m]
Rotor solidity	σ	0.106	[-]
Gravitational acceleration	*g*	9.81	[m/s${}^{2}$]
Air density	ρ	1.225	[kg/m${}^{3}$]
Linear friction coefficient	$k_{t}$	0	[N· s/m]
Angular friction coefficient	$k_{r}$	0	[N · s · m]
Minimum pitch angle	$\alpha_{min}$	0.05	[deg]
Maximum pitch angle	$\alpha_{max}$	15	[deg]
Pitch angle rate limit	$\Delta \alpha_{max}$	60	[deg/s]
Minimum desired square rotor speed	$u_{min}$	0	[rad${}^{2}$/s${}^{2}$]
Maximum desired square rotor speed	$u_{max}$	2 × 10${}^{5}$	[rad${}^{2}$/s${}^{2}$]
Motor square speed rate limit	$\Delta u_{max}$	1.6 × 10${}^{5}$	[rad${}^{2}$/s${}^{3}$]

**Table 2:** Consumptions: independent motors’ speed vs. centralized motor.

Scenario	Consumption (${\left|u\right|}^{3/2}$)
Independent motors	$6.144\times 10^{9}$
Centralized motor	$7.551\times 10^{9}$

## Data Availability

Data sharing not applicable.

## Abbreviations

The following abbreviations are used in this manuscript:

LOE	Loss Of Effectiveness
NDO	Non-linear Disturbance Observer
QP	Quadratic Programming
SQP	Sequential Quadratic Programming
UAV	Unmanned Aerial Vehicle
VPQ	Variable-Pitch Quadrotor
